# Mixed growth of Salix species can promote phosphate-solubilizing bacteria in the roots and rhizosphere

**DOI:** 10.3389/fmicb.2022.1006722

**Published:** 2022-10-20

**Authors:** Piotr Koczorski, Bliss Ursula Furtado, Marcin Gołębiewski, Piotr Hulisz, Dominika Thiem, Christel Baum, Martin Weih, Katarzyna Hrynkiewicz

**Affiliations:** ^1^Department of Microbiology, Faculty of Biological and Veterinary Sciences, Nicolaus Copernicus University, Torun, Poland; ^2^Department of Plant Physiology and Biotechnology, Faculty of Biological and Veterinary Sciences, Nicolaus Copernicus University, Torun, Poland; ^3^Interdisciplinary Centre for Modern Technologies, Nicolaus Copernicus University, Torun, Poland; ^4^Department of Soil Science and Landscape Management, Faculty of Earth Sciences and Spatial Management, Nicolaus Copernicus University, Torun, Poland; ^5^Soil Science, Faculty of Agricultural and Environmental Sciences, University of Rostock, Rostock, Germany; ^6^Department of Crop Production Ecology, Swedish University of Agricultural Sciences, Uppsala, Sweden

**Keywords:** diversity, bacterial endophytes, rhizosphere bacteria, phosphate solubilization, short-rotation cropping, willow diversity, willow

## Abstract

Phosphorus (P) is an essential plant nutrient that can limit plant growth due to low availability in the soil. P-solubilizing bacteria in the roots and rhizosphere increase the P use efficiency of plants. This study addressed the impact of plant species, the level of plant association with bacteria (rhizosphere or root endophyte) and environmental factors (e.g., seasons, soil properties) on the abundance and diversity of P-solubilizing bacteria in short-rotation coppices (SRC) of willows (*Salix* spp.) for biomass production. Two willow species (*S. dasycladoscv*. Loden and *S. schwerinii × S. viminalis cv.* Tora) grown in mono-and mixed culture plots were examined for the abundance and diversity of bacteria in the root endosphere and rhizosphere during two seasons (fall and spring) in central Sweden and northern Germany. Soil properties, such as pH and available P and N, had a significant effect on the structure of the bacterial community. Microbiome analysis and culture-based methods revealed a higher diversity of rhizospheric bacteria than endophytic bacteria. The P-solubilizing bacterial isolates belonged mainly to Proteobacteria (85%), Actinobacteria (6%) and Firmicutes (9%). *Pseudomonas* was the most frequently isolated cultivable bacterial genus from both the root endosphere and the rhizosphere. The remaining cultivable bacterial isolates belonged to the phyla *Actinobacteria* and *Firmicutes*. In conclusion, site-specific soil conditions and the level of plant association with bacteria were the main factors shaping the bacterial communities in the willow SRCs. In particular, the concentration of available P along with the total nitrogen in the soil controlled the total bacterial diversity in willow SRCs. A lower number of endophytic and rhizospheric bacteria was observed in Loden willow species compared to that of Tora and the mix of the two, indicating that mixed growth of *Salix* species promotes P-solubilizing bacterial diversity and abundance. Therefore, a mixed plant design was presented as a management option to increase the P availability for *Salix* in SRCs. This design should be tested for further species mixtures.

## Introduction

Most of the research on plant-bacterial interactions, specifically on woody plant species, focuses either on the general bacterial community diversity or on the role of bacteria in plant interactions, i.e., the bacterial properties that directly affect host plant growth such as plant growth-promoting metabolites, synthesis of phytohormones, and N_2_ binding (Basu et al., 2021). Few studies have addressed bacterial diversity based on their level of association (root endosphere or rhizosphere), and their properties or specific function in woody plants have been scarcely investigated (e.g., Thiem et al., 2018). Meanwhile, in the pool of endophytic and rhizospheric bacteria, there may be those whose direct or indirect influence on plant development is underestimated and not sufficiently investigated to date.

One of the most important contributions of bacteria to plant host growth is their role in facilitating the plant host’s access to available forms of phosphorus (P) (Billah et al., 2019). Several reviews describe the important role of P-solubilizing microorganisms in enhancing overall plant (shoot) growth (Kalayu, 2019; Prabhu et al., 2019; Divjot et al., 2021; Rawat et al., 2021). It is frequently reported that the use of P-solubilizing bacteria (PSB) as bioinoculants is cost-effective and a sustainable alternative to chemical fertilizers, as the excessive use of the latter contributes to lowering the quality of groundwater and soil, as well as the accumulation of toxic elements such as selenium (Se) or arsenic (As) in the soil (Khan et al., 2009; Alori et al., 2017). Experimental studies on the role of P-solubilizing bacteria in plants have mostly used well-known strains of bacteria belonging mainly to *Pseudomonas* sp., *Bacillus* sp. or *Streptomyces* sp. (Wani et al., 2005; Chen et al., 2006; Ahemad and Khan, 2011; Kaur et al., 2011; Grafe et al., 2018; Rathinasabapathi et al., 2018; Ahmad et al., 2019; Wang et al., 2020;). Additionally, screening studies exploring new bacterial strains with P-solubilizing abilities are carried out relatively often (e.g., El Habil-Addas et al., 2017; Rahman et al., 2017; Boubekri et al., 2021; Chen et al., 2021). However, only in some cases are studies on P-solubilizing bacteria conducted on larger scales that take into account (i) the selection and differentiation of strains that are able to degrade easily and sparingly soluble P compounds (belonging to the groups diphosphates and triphosphates, respectively), (ii) the compatibility of specific groups of P-solubilizing bacteria with specific plant species and specific environmental conditions and cultivation systems (e.g., monoculture or mixed cultivation), and (iii) the application potential of these microorganisms. High-throughput methods such as metagenomic analysis, which can provide the background of the entire community of bacteria of a given environment, are also rarely targeted by the previously mentioned research (Sarikhani et al., 2019; Teng et al., 2019; Chawngthu et al., 2020; Rfaki et al., 2020). Meanwhile, the evaluation of the microbiological background of the microorganisms coexisting with P-solubilizing microbes is extremely important because the composition of the microbiological background can be used as a guide for selecting the right bacteria that will be compatible not only with a specific plant species but also with its microbiome.

Our work responds to the above deficiencies and presents not only the screening of cultivable P-solubilizing bacteria (e.g., di-and triphosphates) but also a metagenomic analysis of the rhizosphere and root endosphere, which provides a proper background of the bacterial community. The research was carried out in experimental trials including single-species and mixed-species plots of two willow varieties belonging to different species (Loden (*S. dasyclados*) [L] and Tora (*S. schwerinii × S. viminalis*) [T]), which were grown in two locations with different soil and climatic conditions (Germany and Sweden). This study focused on bacteria and complements our previous results on P-solubilizing fungi in the same experimental trials (Koczorski et al., 2021). The main objective was to assess the influence of soil properties, seasons, the level of plant association with bacteria (root endophyte and rhizosphere), and the host plant cultivation system (single-species vs. mixed-species) on the diversity of P-solubilizing bacteria and to determine their role and contribution to the background of the entire willow microbiome. The application of culturable and nonculturable research techniques allowed us to evaluate the culturable PSB in the context of a complete community of bacteria, as well as to determine the influence of soil properties and seasons on changes in community structure. We hypothesized that (i) bacteria with the ability to solubilize P would be more abundant in the rhizosphere than the root endosphere of the tested plants and (ii) the plant species and the test site conditions would significantly affect the bacterial diversity and bacterial community.

## Materials and methods

### Sample collection and processing

**Figure 1:**
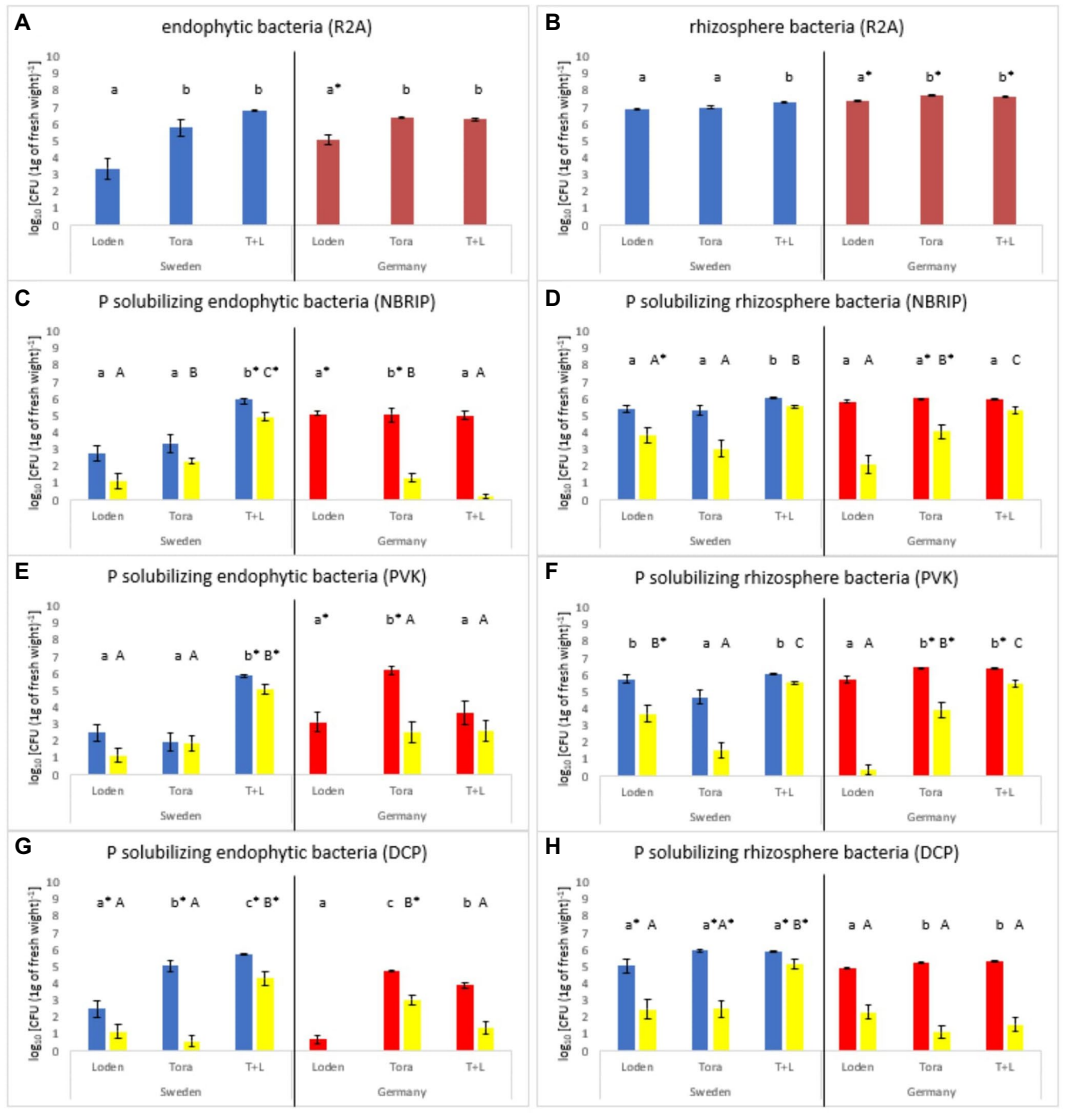
Total **(A,B)** and P-solubilizing bacteria **(C–H)** abundance on different media. Samples were taken in fall 2018 from two test sites (Sweden and Germany). **(A,B)** present the total bacterial count on plates with R2A medium for two levels of plant associations (rhizosphere and endophytic bacteria). Blue bars represent samples from Sweden and red from Germany. **(C–H)** show the abundance of bacteria present on selective media plates (blue and red bars) and the abundance of P-solubilizing bacteria (yellow bars). Small and capital letters indicate significant differences between the two willow species used (Tora = *S. schwerinii x viminalis*, Loden = *S. dasyclados*) (small letters for total bacteria count; capital letters for P-solubilizing bacteria). ^*^Indicates differences between sites.

The test sites used in this study are two experimental trials of willow short-rotation coppices (SRCs) located in Uppsala (Sweden: 59.820375, 17.640334) and Rostock (Germany: 54.061391, 12.081857). Both sites were previously used as arable sites, and they differ in the soil type. The soil at site S is characterized as a Vertic Cambisol type (moderate amount of clay), while at site G, the soil type is described as a Stagnic Cambisol (sandy loam dominance) (IUSS Working Group WRB, 2015). The experiment was established in 2014 as part of the ECOLINK-Salix to investigate the impact of willow diversity on growth, nutrient use and ecosystem functioning (Hoeber et al., 2018). A detailed description of the experimental trials is found elsewhere (Hoeber et al., 2018; Koczorski et al., 2021; Figure 1). In our investigation, we only used the data from two willow genotypes belonging to two different species, Loden (*S. dasyclados*) and Tora (*S. schwerinii* × *S. viminalis*), and compared them between the two test sites (German and Swedish). Tora is characterized by a higher growth rate and smaller total leaf area than Loden (Hoeber et al., 2018). At each test site, three blocks (replicates) were established, each consisting of three plots with willows planted in single-species or mixed-species cultures. The size of each plot was 9.6 × 9.6 m, and the tree planting density was 15,600 plants per ha (Hoeber et al., 2018). Samples for analysis were collected at both sites in two seasons: fall (October) 2018 and spring (May) 2019. During each season, samples from each experimental site were collected from three plots in three replicates: Loden monoculture, Tora monoculture and mixed cultivation of both (Loden and Tora). In total, 162 samples (81 from Sweden and 81 from Germany) of willow roots and adjacent soils (15 × 15 × 15 cm) sampled at equal intervals of 6 m from each other were collected. The collected samples were immediately transported to the laboratory in Poland (Department of Microbiology, Nicolaus Copernicus University) and analyzed as described below. The samples collected in fall 2018 were used to determine the total number of bacteria and the total number of P-solubilizing bacteria (PSB), for which a collection was also established. The roots were carefully separated from adherent soil and subjected to a surface sterilization process as described previously (Koczorski et al., 2021). In the first step of surface sterilization, 60% alcohol was used (3 min), and then the roots were rinsed 3 times in a sterile 2% NaCl solution. In the second step, 5% H_2_O_2_ solution (10 min) was used for sterilization, and roots were again rinsed 3x in sterile 2% NaCl solution. The solutions from the last rinse were used to perform sterilization control on R2A medium (Difco, United States).

### Soil analysis

In this study, soil data obtained in previous studies were used (Koczorski et al., 2021; Supplementary Table S1). Soil samples were sieved through a 2-mm-mesh screen after air-drying for the following analyses: total organic carbon (TOC) and total nitrogen (TN) contents were measured after dry combustion using a CHNS Vario Macro Cube elemental analyzer. Available phosphorus (P_av_) in 1% citric acid (Van Reeuwijk, 2002) was determined by a spectrophotometric method using a UV–Vis Rayleigh UV-1601 spectrophotometer (Van Reeuwijk, 2002), and the pH at a 1:2.5 soil to water ratio was determined by the potentiometric method using an Elmetron CP-105 pH meter.

### Isolation of endophytic and rhizospheric total culturable bacteria and screening for P-solubilizing bacteria

Sterile roots (1 g) were homogenized in a sterile mortar and transferred to 9 ml of a sterile 0.5% NaCl solution. The processed rhizosphere soil samples (1 g) were transferred to 9 ml of a sterile 0.5% NaCl solution. Serial dilutions (10^−1^ – 10^−7^) were prepared and used for inoculations (spread plating technique) on R2A media with the addition of 40 mg/μl nystatin (for roots: 10^−3^ and 10^−4^, for rhizosphere soil: 10^−4^ and 10^−5^) in 3 replicates for each variant and dilution (108 Petri plates, in total). The plates were incubated for 7 days at 24°C, and then total bacterial counts were determined and presented as colony-forming units (cfu).

Three selective media were used for the isolation of phosphate (P)-solubilizing bacteria: NBRIP, PVK containing tri-phosphates (Nautiyal, 1999) and DCP containing di-phosphates (modified by Pikovskaya, 1948) (composition of media presented as Supplementary materials in Koczorski et al., 2021).^1^ The inoculations were made from the same serial dilutions (mentioned above) that were used to determine the total number of bacteria. For NBRIP and PVK media, dilutions of 10^−6^ and 10^−7^ were used (roots and rhizosphere soil), and for DCP media, dilutions of 10^−4^– 10^−5^ (roots) and 10^−5^ – 10^−6^ (rhizosphere soil) were used. All analyses were performed in three technical replications (324 plates in total, 108 plates for each of the selective media). After 7 days of cultivation at 24°C, colonies with visible halo zones were noted. The presence of halo zones indicated the P-solubilizing ability of the isolated strains and allowed for the selection of positive strains for further analysis.

In the experiment, the total number of P-solubilizing bacteria and the total number of bacteria grown on the tested media were determined to assess the potential of the investigated sites and zones (rhizospheric and endophytic) for colonization by potential P-solubilizing bacteria. The bacterial isolates with the ability to solubilize P on selective media were selected and transferred to R2A medium for further identification using molecular methods.

### Identification of culturable P-solubilizing bacterial strains

A Bacterial and Yeast Genomic DNA Purification Kit (EurX, Poland) was used to isolate bacterial DNA. The isolated DNA was quantified using a UV–Vis spectrophotometer (NanoDrop 2000, United States). Bacteria were identified based on the 16S rRNA region using primers 27f (5’-AGAGTTTGATCMTGGCTCAG-3′) and 1492r (5’-TACGGYTACCTTGTTACGACT-3′; Frank et al., 2008). The PCR products were purified using the PCR/DNA Clean-Up Purification Kit (EurX). A 1% agarose gel (1 x TBE buffer) with the addition of Simply Safe (EurX) dye was used to confirm the presence of PCR products after purification. The length of the PCR products was determined based on the 100 bp ladder (Perfect 100 bp DNA Ladder, EurX). The genetic material was sequenced at the Institute of Biochemistry and Biophysics.^2^ Sequencher 5.4.6 software was used to prepare contigs, and the obtained sequences were compared with those in the NCBI database using BLASTn, which is available on the National Center for Biotechnology Information (NCBI) servers.^3^ Mega X software (Kumar et al., 2018) was used for phylogenetic analysis according to the procedure described by Furtado et al. (2019). Reference sequences were obtained from NCBI, and phylogenetic analysis was performed using the neighbour-joining method. Evolutionary distances were determined with the use of the p-distance method (Saitou and Nei, 1987). The phylogenetic tree was visualized with Interactive Tree of Life (iTOL) v3 (Letunic and Bork, 2016).

### Statistical analysis

Statistical analyses were performed using Statistica software (version 13.0, StatSoft). Mean values and standard deviations were calculated. The computed data from both the isolation of total culturable bacteria and screening for culturable P-solubilizing bacteria are presented as Colony-Forming Unit (CFU)/g dry weight.

### Assessing nonculturable bacterial diversity

Total DNA (from the roots and rhizosphere) was isolated according to Koczorski et al. (2021). DNA was isolated from 50 mg of lyophilized roots (for endophytic diversity) and 50 mg of air-dried rhizosphere soil, and each sample was prepared in 3 replicates. Root samples were homogenized with plastic beads prior to isolation. The spectrophotometric (NanoDrop 2000, United States) and fluorometric (Qubit 2.0) methods were used to determine the concentration of DNA in the samples. DNA prepared in this way was used to create libraries in a two-step PCR and purified using Agencourt AMPure XP (Beckman Coulter). The quality and quantity of libraries were assessed using the Bioanalyzer chip (Agilent) and the KAPA Library Quantification Kit for the Illumina Platform – LightCycler 480 (Roche). The complete process of DNA isolation and library preparation was described in our previous work (Thiem et al., 2018), and the method of statistical analysis was described in the publication by Koczorski et al. (2021).

## Results

### Culturable bacteria and their phosphate solubilization activity at two sites

The number of culturable bacteria ranged between 3.5 and 6.8 cfu/g of dry roots for endophytes and 6.89–7.89 cfu/g of dry soil for the rhizosphere. In general, the tests showed that there was a significant difference in endophytic bacterial abundance between the tested variants of the experiment (mono and mixed cultures). In the case of the willow species, a lower number of endophytic and rhizospheric bacteria were observed in Loden than in both Tora and the mix of both (Loden and Tora; Figure 1). The difference between P-solubilizing bacterial abundance in the Loden species and the mixed variant was an almost twofold lower, indicating the importance of Tora species presence. The NBRIP and PVK selective media showed very similar trends for Loden, where the frequency of occurrence of isolates with phosphate solubilization was lower than in the other tested variants (single-species Tora and the species mix). At the Swedish site, the mixed cultures showed the highest number of PSB both in the case of endophytic and rhizospheric bacteria, while at the German site, the number of PSB was the highest in Tora for endophytic and the mixed culture for the rhizospheric bacteria (Figure 1). Loden showed a lower number of PSBs associated with the plant (both endophytic and rhizospheric bacteria). Overall, the NBRIP medium selected more PSBs from the Swedish site, while in the PVK medium, the PSB abundance was higher in the German site. The total abundance of PSB on the DCP medium was lower than that on the NBRIP and PVK media. The number of PSBs on the DCP medium was lowest for Loden at both experimental sites and both levels of association with the plant (endophytes and rhizosphere). For endophytes in the Swedish site, the mixed cultures showed the highest number of PSB, while for the German site, it was Tora. Similarly, for the rhizosphere, the PSB was the highest for the mixed cultures in the Swedish site, while no significant differences were observed in the German site.

### Identification of culturable phosphate-solubilizing bacteria

**Figure 2:**
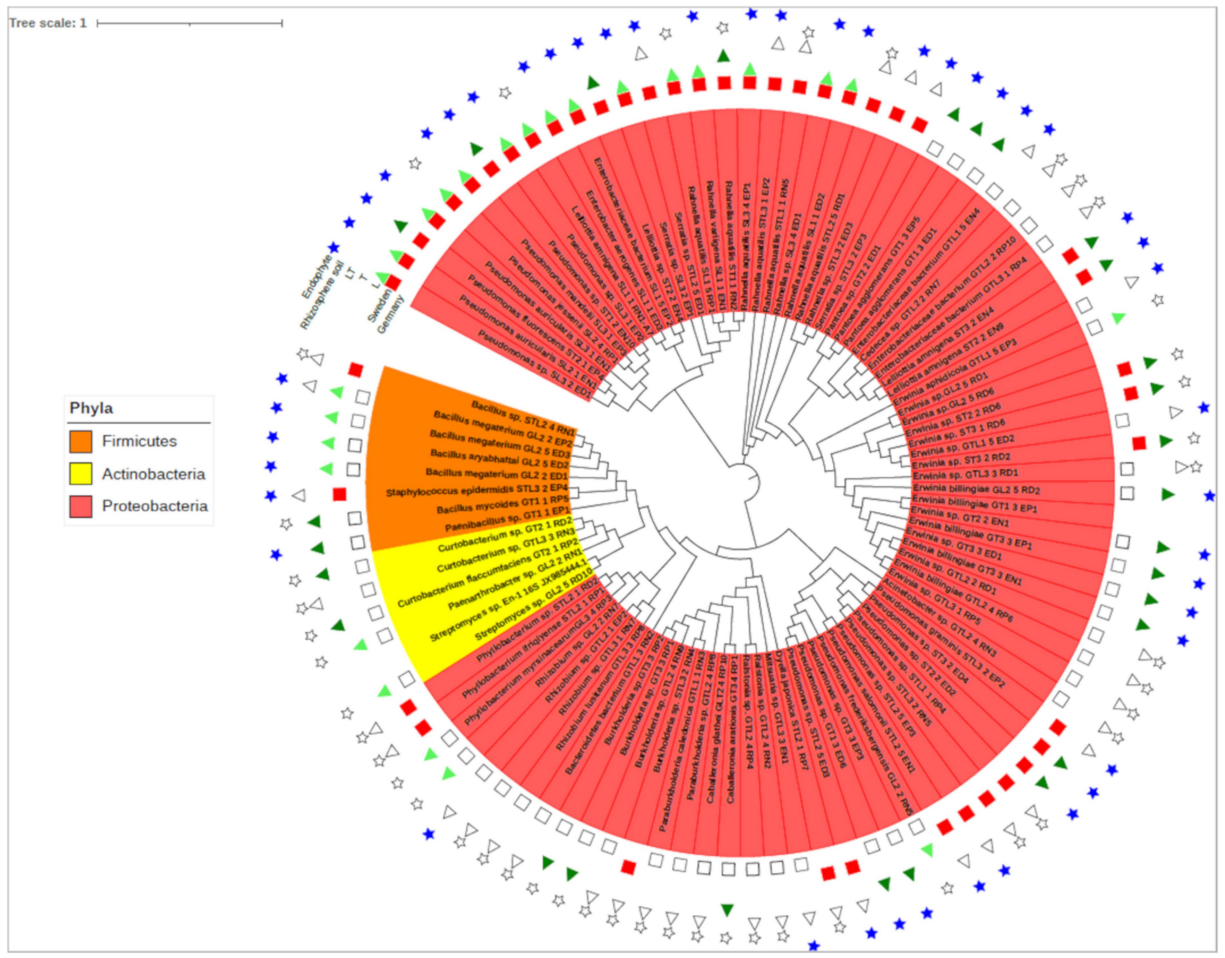
Phylogenetic analysis of culturable bacteria isolated from two willow species and their mixture classified at the phyla level. For details of isolates with their GenBank accession numbers, see Supplementary Table S1B. Reference sequences with the closest BLAST match were used (strains without symbols) to construct the phylogenetic tree.

During the experiment, 88 different bacterial strains with the ability to solubilize P were isolated. Among this bacterial collection, 61 were isolated from tri-phosphate-containing media (26 from NBRIP, 35 from PVK) and 27 from di-phosphate (DCP) media. A total of 41 different endophytic and 47 rhizospheric strains were isolated. Almost 85% of the isolated bacteria (74 strains) belonged to *Proteobacteria*, 6 belonged to *Actinobacteria* (6%) and 8 belonged to *Firmicutes* (9%). The dominant taxa among *Proteobacteria* included species of *Pseudomonas* with 12 strains (13%), *Erwinia* with 17 (19%) and *Rahnella* with 10 (11%). *Actinobacteria* was dominated by the genus *Curtobacterium,* with 3 identified strains (50%), and *Firmicutes* was dominated by 6 strains from the *Bacillus* (67%) genus. A total of 51 strains with the ability to solubilize P at the German site and 37 at the Swedish site were obtained. At the German site, more rhizospheric than endophytic PSBs were isolated, while at the Swedish site, we observed the reverse trend. The most frequently isolated PSB at the Swedish site were bacteria belonging to the genera *Pseudomonas* and *Rahnella*, both found in the endosphere and the rhizosphere. The genus *Erwinia* was characteristic of the German site for both levels of association with the plant (endosphere and rhizosphere). The highest number of strains belonging to *Actinobacteria* were isolated from the German site. Almost 50% of the strains were isolated from the mixed culture (LT; Figure 2).

### Microbiome analysis

**Figure 3:**
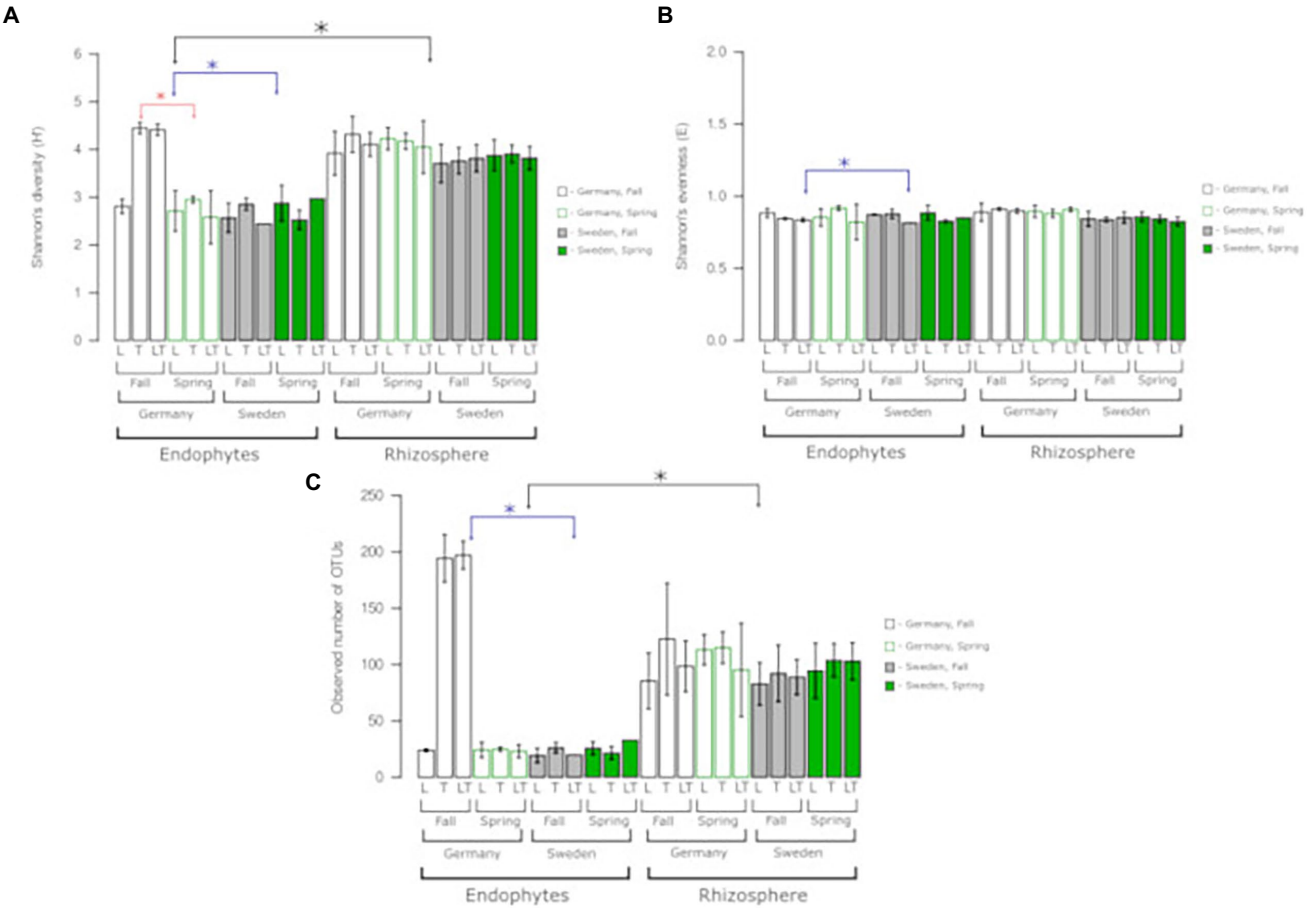
Bacterial species richness, diversity, and evenness across two experimental sites, rhizosphere soil and roots. OTUs were constructed at 0.03 dissimilarity for bacterial sequences. **(A)** Shannon’s (H), **(B)** Shannon’s (E), **(C)** observed number of OTUs. Robust ANOVA with Tukey’s post-hoc analysis was used to assess the significance of differences between experimental sites, rhizosphere soil, and roots. Colours denote the following: black – significant difference between endophytes/rhizosphere, blue – significant differences between sites, red-significant difference between seasons. ^∗^Indicates a significant difference between variants indicated by arrows.

The bacterial alpha diversity was mainly determined by the level of association with the plant (endosphere and rhizosphere), whereas in the case of endophytes, it was additionally determined by the experimental sites (S, G; Figures 3A–C). A significant difference between the rhizospheric and endophytic bacterial diversity was found in the Shannon’s H′ and the number of OTUs observed, wherein the bacterial population in the rhizosphere soil was more diverse (Figure 3A). In the case of endophytic bacteria at site G, significant differences were observed between the cultivable variants and the seasons. This is due to the high values obtained for the samples from the Tora mono-and mixed cultures, which differed significantly from those of the other variants (Figure 3A). The analysis of the Shannon evenness index showed a significant difference between the endophytic communities from the German and Swedish sites (Figure 3B). No significant differences were observed in other variants of the experiment (Figure 3B). The number of observed OTUs showed a significant difference between endophytes and the rhizosphere, as well as between the sites within the endophyte variant (Figure 3C). Overall, the most prominent difference in the diversity of the bacterial microbiome was observed for endophytes and is related to the differences between the studied sites (Swedish and German) Shannon’s H′, Shannon’s E and the number of observed OTUs.

### NMDS and CCA analysis

**Figure 4:**
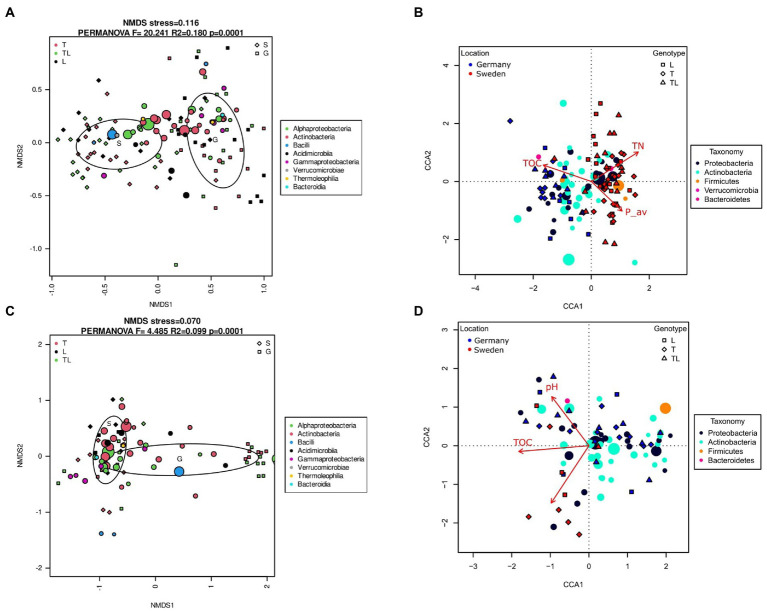
Analysis of log-transformed and Wisconsin double-standardized Bray–Curtis dissimilarity matrix for rhizosphere soil and root bacterial communities associated with *Salix* species L (Loden = *S. dasyclado*s), T (Tora = *S. schwerinii x viminalis*), and LT (mixture) between two seasons (fall, spring) and two sampling sites (Germany and Sweden). **(A,C)** NMDS (nonmetric multidimensional scaling analysis); **(B,D)** CCA (canonical correspondence analysis); **A,B** plots – Soil; **C,D** – Root samples.

In this study, soil data obtained in previous studies were used (Koczorski et al., 2021; Supplementary Table S1). According to the results of NMDS analysis, the bacterial communities in the rhizosphere (Figure 4A) and endosphere (Figure 4C) were clustered based on sites (S and G). A PERMANOVA test confirmed that the grouping of rhizosphere and endosphere variants was statistically significant (rhizosphere soil: *F* = 20.241 *r*^2^ = 0.180 *p* = 0.0001 roots: *F* = 44.85 *R*^2^ = 0.099 *p* = 0.0001). Grouping of bacterial communities by season was not observed. The samples from the German site were more scattered, especially from the roots, indicating higher variability at this test site. The separation between sites was much more prominent in the case of the rhizosphere, which suggests that these communities show greater differentiation than the endophytic communities. NMDS ordination showed clear division of samples from the German site according to willow species, with Loden grouped with Sweden samples, while Tora and mixed variants remained grouped together (Figure 4C). The samples from Tora and the mixed cultures were separated from the Loden samples that were grouped with the samples from the Swedish site. The CCA confirmed the separation of samples from the rhizosphere by site. In Figure 4B, the total nitrogen (TN) and partially available phosphorus (P_av_) were the key factors responsible for the separation of samples with a positive correlation for site S. The rhizosphere samples were more scattered and formed 3 separate groups. The first group correlated with P_av_ and consisted mainly of samples from the Tora and mixed cultures (TL) from Sweden. The second group showed a high correlation with pH (pH_water) and was composed of the samples belonging to Tora and mixed cultures (TL) from Germany. The remaining samples that formed the third group showed a negative correlation with all the previously mentioned factors and with organic carbon (TOC). CCA analysis revealed that for soil samples, the phylum *Firmicutes* showed a positive correlation with P_av_, while *Proteobacteria* correlated with TN. For root samples, there was no clear correlation with any of the bacterial strains.

### Bacterial diversity at different taxonomic levels

**Figure 5:**
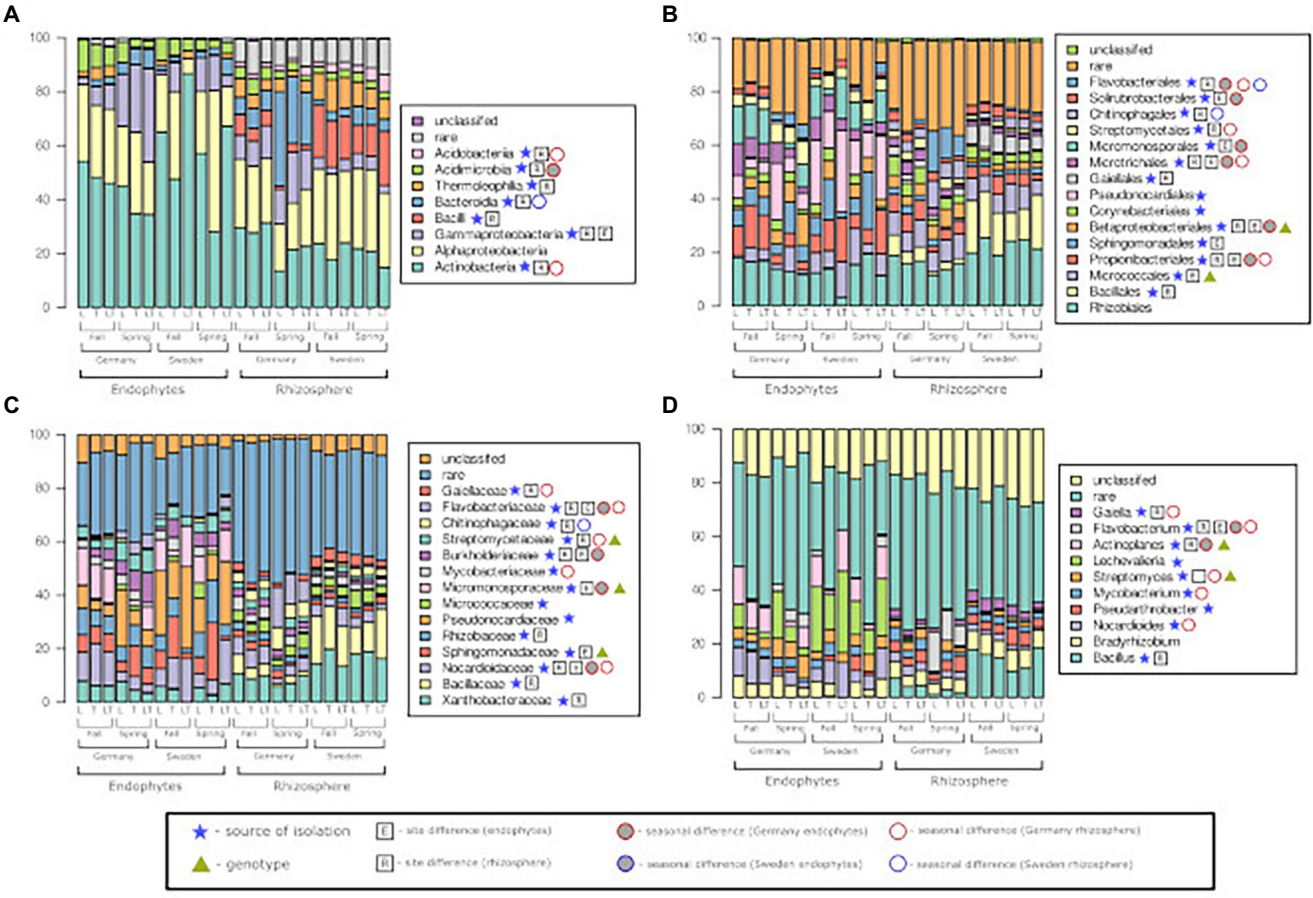
Bacterial community structure at the level of class **(A)**, order **(B)**, family **(C)**, and genus **(D)**, among rhizosphere soil and roots, two test sites and two seasons.

As with the identification of isolated bacteria, most of the reads obtained in the microbiome analysis belonged to the phyla *Actinobacteria* and *Proteobacteria*, especially in the communities of endophytic bacteria from Sweden (Figure 5A). At the class level, significant differences were also observed between the level of bacterial association and the location of the test site. Additionally, some seasonal differences were observed, but only for samples from site G. At the class level, no differences between the two willow species were observed (Figure 5A). At the order level (Figure 5B), we observed a high number of different taxa across all samples. Most of the samples showed significant differences in the level of plant association (endophyte, rhizosphere) and site (Figure 5B). Bacterial community analysis showed that similar to the class level (Figure 5A), a small number of samples showed differences between seasons. However, in the case of the samples from Sweden, differences between the willow species were observed. Figures 5C,D show that this trend is also valid for the remaining taxonomic levels (family and genus). It was also shown that the lower we are at the taxonomic level, the higher the number of reads belonging to the ‘rare’ category (number of reads is less than a predetermined threshold).

## Discussion

The aim of this work was to gain insight into bacterial diversity and to identify P-solubilizing bacteria in the rhizosphere and endosphere of single-species and two-species plots of short-rotation willow coppices. Moreover, this is also an extension of a previous investigation that aimed at isolating and identifying P-solubilizing microorganisms (fungi) from the same experimental trials and comparing them with the results of the microbiome analysis (season comparison included; Koczorski et al., 2021). In the present work, we expected that the increased diversity of host plant species grown in the species mixture may favor a greater diversity of microorganisms, including phosphate-solubilizing microorganisms. Consequently, biomass production may be enhanced (Chen et al., 2021). To verify this, we used a classical microbiological approach combined with NGS analysis. The results presenting the number of P-solubilizing bacteria revealed that there was a significantly higher number of bacteria in the rhizosphere than in the endosphere. It could be that only selected bacterial strains can penetrate the root’s intercellular space and form endophytes. Most plant species are known to produce substances that either attract or inhibit microbial root entry (Jacoby et al., 2017). As such, the roots can act as a filter through which only selected bacteria can penetrate and form a well-functioning symbiosis with the host plant (Taulé et al., 2021). Moreover, bacteria that form endophytes are known to possess specific properties or functions that help to survive in plant tissues and establish this symbiosis (Papik et al., 2020). Furthermore, the results from the culturable diversity showed the lowest number of P-solubilizing bacteria for Loden species (*S. dasyclados*) grown in monoculture and the highest number in the case of the species mixture (at both sites and levels of association of microorganisms with the plant: endo-and rhizosphere). Therefore, we can expect that an increased number of plant species in a cultivation system will promote microbial diversity and function. This suggests that both species identity and the cultivation of mixed species at the same site can have positive effects on the number of P-solubilizing bacteria. The mixing effect may be due to the higher competition of microorganisms for nutrients in mixed cultures, which promotes the diversity of rhizospheric and endophytic microorganisms (Weih et al., 2019). A similar conclusion was made by Schweier et al. (2019), where mixed cultures of willow species and genotypes produced higher biomass and positively influenced the diversity of microorganisms at the site.

The soil analysis presented in our earlier publication showed a much higher level of P at the Swedish site than at the German site (Koczorski et al., 2021). This may suggest that the increased P availability in the soil has a positive effect on the abundance of PSBs (rhizosphere and endophytes). Our experiment showed the presence of bacteria that were able to grow on media without available P but at the same time did not show any visible signs of P solubilization (halo zones). Since the bacteria were grown on the media for 7 days, it is possible that their cells had some reservoir of P that allowed them to grow. Liu et al. (2015) made similar observations using media supplemented with the same P source as we did, and they stated that lack of clear signs of P solubilization might also be a result of their very low activity. The most frequently isolated PSB in our experiment were *Pseudomonas*, *Erwinia* and *Bacillus.* These genera are well known and frequently isolated as PSBs, especially *Pseudomonas* (Yu et al., 2011; Sarker et al., 2014) and *Bacillus* (Liu et al., 2015; Chawngthu et al., 2020). These strains have found applications as biofertilizers for the cultivation of wheat (Liu et al., 2019), maize (Viruel et al., 2014), rice (Gomez-Ramirez and Uribe-Velez, 2021) and fruit trees, e.g., apple trees (Kurek et al., 2013). *Erwinia* sp. is often mentioned in the literature as a plant pathogen; however, the *Salix* trees grown at the two test sites did not show any symptoms of infection during sampling. Few researchers have shown the potential of *Erwinia* sp. as a plant growth-promoting bacterium and confirmed its presence in underground and aboveground parts of plants, e.g., almond trees (Guzmán et al., 2021), apple trees, pear trees (Rezzonico et al., 2016) and barley (Li et al., 2021). This suggests that willow trees are a potential source of both well-known and completely new P-solubilizing microorganisms. Our work also provides insight into P-solubilizing bacteria that can potentially be used as a part of specific willow SRC biofertilizers.

The Shannon diversity index (H′) and the number of observed OTUs confirmed that the bacterial diversity of the rhizosphere was higher than that of the endosphere. The same trend was observed by Tardif et al. (2016), where alpha diversity was also higher in the rhizospheric than endophytic bacteria. Additionally, we observed a very large difference between the root endophytes derived from site G of the single-species Tora plots and the mixed-species plots, which showed much higher diversity than the rhizosphere samples. This is in agreement with the results obtained from the culturable bacterial abundance assessment on R2A medium, where Loden showed significantly lower numbers of bacterial isolates than Tora and the mixed plots. This result suggests that there is a strong connection between host plant species and microbial community composition in the rhizosphere and the endosphere.

In our previous publication that describes the abundance and diversity of fungi, we observed differences in alpha diversity for sites and levels of association with the plant, while no differences between host plant species were seen (Koczorski et al., 2021). In comparison to the fungal diversity, the bacterial diversity analysed using Shannon’s index (H′) was higher than that of fungi with 4.5 (bacteria) and 3 (fungi) for rhizosphere soil and 3 (bacteria) and 2.5 (fungi) for endophytes. The NMDS analysis showed a clear division of samples according to the test sites, except for endophytic bacteria at site G, where a statistically significant separation between genotypes was observed. The separation of samples by the two sites in the NMDS analysis for fungi was more distinct, and the samples showed a greater concentration (Koczorski et al., 2021). This suggests that fungal communities are more dependent on soil and weather conditions or nutrient content, while bacteria are mostly dependent on pH and soil structure (Lauber et al., 2008; Furtado et al., 2019).

The results of the CCA analysis showed that among the assessed variables, total nitrogen and available P were the main factors driving bacterial community diversity, while for fungi, total nitrogen and organic carbon were the main factors driving community diversity (Koczorski et al., 2021). The high similarity of samples from the Tora monoculture and the mixed cultures at site G with soil pH suggests that the increase in bacterial diversity could be caused by the higher pH of the soil at this site. The significant effect of pH on bacterial diversity was analyzed by Tripathi et al. (2018), and their results indicate that any deviation from neutral pH significantly affects the structure of soil microorganisms. Furthermore, the research conducted by Kuzovkina et al. (2018) showed a significant positive effect of higher soil pH on the growth of willow, which may also increase the diversity of microorganisms by promoting P-solubilizing microorganism abundance. Although acidification is a strong indicator of P solubilization, we did not observe a positive correlation between those two factors in our analyses. The optimal pH for P solubilization appears to range from 5.5 to 7.5 (Alori et al., 2017), and at both of our sites, the pH was in the reported range. This indicates that there are other factors that can influence P solubilization efficiency.

For the bacterial community, the dominance of *Actinobacteria* and *Alphaproteobacteria* in the soil environment and willow roots was also reported by Tardif et al. (2016) and Yergeau et al. (2015). However, it should be mentioned that these studies were conducted in contaminated areas that are usually dominated by *Proteobacteria*. In the abovementioned studies, as in our work, there was a difference in the bacterial community between the endo-and rhizosphere, which was evident in the significant differences in the abundances of *Actinobacteria*, *Alphaproteobacteria* and *Firmicutes*. Bacteria belonging to 10 different genera were observed. Among them, we found possible P solubilizers. *Bacillus* is a genus reportedly comprising many P-solubilizing organisms. They are also known for their antifungal properties and IAA and ACC deaminase synthesis (Cherif-Silini et al., 2016). Another efficient P-solubilizing genus of bacteria found in willows is *Streptomyces*. They are also known for their antifungal properties (Cao et al., 2004; Khamna et al., 2009; Jog et al., 2014). Other, not as explored, genera of P solubilizers found in our experiment are *Gaiella* and *Nocardioides* (Albuquerque et al., 2011). *Nocardioides* was also reported to possess the ability to degrade casein and Tweens 20, 40 and 80 (Roh et al., 2020). Additionally, in Figure 5D, it can be observed that bacteria from the genus *Bradyrhizobium* do not differ significantly between the variants, which suggests that they belong to the group of microorganisms characteristic of willow regardless of the tested variants of the experiment. Other genera found in our study were also reported and are briefly described in Supplementary Table S1A in the supplementary materials. The collection of isolated and characterized P-solubilizing bacteria will be used in our future experiments to select the most efficient P-solubilizing bacteria in short-rotation coppices of willow.

## Conclusion

The level of plant association (direct: endophytes vs. indirect: rhizosphere contact with plant tissue) is the most important factor shaping the bacterial community in willow short-rotation coppices, with dominance of Actinobacteria in the endosphere and Gammaproteobacteria and Bacilli in the rhizosphere. Cultivation of a mixed willow species system increases the abundance of P-solubilizing bacteria, which can ultimately minimize the problem of low P availability in agricultural soils and lower the need for fertilizer application.

## Data availability statement

The datasets presented in this study can be found in online repositories. The names of the repository/repositories and accession number(s) can be found below: https://www.ncbi.nlm.nih.gov/bioproject, PRJNA716888; https://www.ncbi.nlm.nih.gov/genbank/, OP102593- OP102680.

## Author contributions

PK participated in all analyses and wrote the first version of the manuscript. BF participated in preparation of manuscript. MG designed the bioinformatics pipeline, performed bioinformatics analyses, and participated in the preparation of the manuscript. DT participated in manuscript preparations and total DNA isolations and prepared libraries required for sequencing. PK and BF analyzed the results and the statistical output. PK, CB, and MW performed sampling at the locations, selected plant genotypes for the experiments, and provided input to the manuscript. PH did soil analyses and participated in the preparation of the manuscript. KH designed and managed the field and lab experiments and participated in the preparation of the manuscript. All authors contributed to the article and approved the submitted version.

## Funding

The establishment and management of the Swedish field trial was funded by grants from the Swedish Energy Agency (project nos. 36654-1 and 36654-2). Parts of the research in the Swedish trial were also funded by The Swedish Research Council Formas (project no. 942-2016-31). All microbiological and molecular analyses as well as manuscript editing were funded from the project: Universitas Copernicana Thoruniensis In Futuro – modernization of the Nicolaus Copernicus University as part of the Integrated University Program (project no. POWR.03.05.00-00-Z302/17-00) implemented under the Knowledge Education Development Operational Program.

## Conflict of interest

The authors declare that the research was conducted in the absence of any commercial or financial relationships that could be construed as a potential conflict of interest.

## Publisher’s note

All claims expressed in this article are solely those of the authors and do not necessarily represent those of their affiliated organizations, or those of the publisher, the editors and the reviewers. Any product that may be evaluated in this article, or claim that may be made by its manufacturer, is not guaranteed or endorsed by the publisher.

## Footnotes

^1^ https://www.frontiersin.org/articles/10.3389/fpls.2021.647709/full

^2^ http://oligo.ibb.waw.pl/

^3^ http://www.ncbi.nlm.nih.gov/BLAST
