# Supplementary material for: Mixed growth of Salix species can promote phosphate-solubilizing bacteria in the roots and rhizosphere
Source: Front Microbiol. 2022 Oct 20;13:1006722. doi: 10.3389/fmicb.2022.1006722 (PMC9634750; doi:10.3389/fmicb.2022.1006722)
Supplement: Supplementary file 1 [file Data_Sheet_1.docx]

**Mixed growth of *Salix* species can promote phosphate-solubilizing bacteria in roots and rhizosphere**

Piotr Koczorski^1^, Bliss Ursula Furtado^1^, Marcin Gołębiewski^2,3^, Piotr Hulisz^6^, Dominika Thiem^1^, Christel Baum^4^, Martin Weih^5^, Katarzyna Hrynkiewicz^1*^

^1^Department of Microbiology, Faculty of Biological and Veterinary Sciences, Nicolaus Copernicus University, Torun, Poland

^2^Department of Plant Physiology and Biotechnology, Faculty of Biological and Veterinary Sciences, Nicolaus Copernicus University, Torun, Poland

^3^Interdisciplinary Center for Modern Technologies, Nicolaus Copernicus University, Torun, Poland

^4^Soil Science, Faculty of Agricultural and Environmental Sciences, University of Rostock, Rostock, Germany

^5^Department of Crop Production Ecology, Swedish University of Agricultural Sciences, Ullsväg 16, 75007, Uppsala, Sweden

^6^Department of Soil Science and Landscape Management, Faculty of Earth Sciences and Spatial Management, Nicolaus Copernicus University, Torun, Poland

**Table A** Characterization of bacteria present in microbiome analysis on genus level.

| **Bacteria genus** | **Group** | **Source of isolation** | **Potential function** | **References** |
| --- | --- | --- | --- | --- |
| *Gaiella* | ND | Deep mineral water | Acid and Alkaline phospohatese activity | Albuquerque et al., 2011 |
| *Flavobacterium* | Rhizosphere/ endophyte | Rhizosphere e.g. soybean/ maze roots | Intracellular α-gal, α -glu and α-glu activities.  Catalase- and oxidase-positive  Nitrogene fixation | Mawdsley, & Burns 1994  Madhaiyan et al., 2010  Gao et al., 2015 |
| *Actinoplanes* | rhizosphere | Rhizopshere of coconut tree | Cellulase production | Luo et al., 2021 |
| *Lechevalieria* | Soil | Desert soil | Catalase production, acid production from various substanes (e.g. Lactose, Mannitol, Mannose, Salicin, Trechalose) | Okoro et al., 2010 |
| *Streptomyces* | Rhizosphere/ Endophytes | Rhizosphere of Thai medicin plants and wheat/ Wheat seeds | Antifungal, P solubilization | Khamna et al., 2009  Jog et al., 2014  Cao et al., 2004 |
| *Mycobacterium* | Rhizosphere | Rhizosphere of Barley | Mineralization of Pyrene | Egamberdieva 2012 |
| *Pseudoarthrobacter* | Soil | Crop fields | Biodegradation of syntetic dyes | Stedel et al., 2019 |
| *Nocardioides* | Soil | Sandy soil | Casein,Tweens 20, 40 and 80 degradation and Alkaline phosphatase activity | Roh et al., 2020 |
| *Bradyrhizobium* | Endophyte | Roots of Sorghum | Nitrogene fixation | Hara et al., 2019 |
| *Bacillus* | Rhizosphere | Wheat rhizosphere | Antifungal properties,production of IAA and ACC deaminase, P solubilization and | Cherif-Silini et al., 2016 |

**Table B** P solubilizing bacterial identification table with Accession numbers. S- Sweden, G- Germany, L – Loden, T – Tora, LT – mixture, E – endophyte, R – rhizosphere bacteria

| **Symbol** | **Identified as** | **Country** | **Genotype** | **Place of isolation** | **Accession number** |
| --- | --- | --- | --- | --- | --- |
| B1 | *Lelliottia amnigena* | S | L | R | OP102593 |
| B2 | *Rahnella aquatilis* | S | L | R | OP102594 |
| B3 | *Rahnella aquatilis* | S | T | R | OP102595 |
| B4 | *Enterobacter aerogenes* | S | L | E | OP102596 |
| B5 | *Rahnella variigena* | S | L | E | OP102597 |
| B6 | *Serratia* sp. | S | L | E | OP102598 |
| B7 | *Rahnella aquatilis* | S | L | E | OP102599 |
| B8 | *Pseudomonas mandelii* | S | L | E | OP102600 |
| B9 | *Pseudomonas frederiksbergensis* | G | L | R | OP102601 |
| B10 | *Erwinia* sp. | G | L | R | OP102602 |
| B11 | *Streptomyces* sp. | G | L | R | OP102603 |
| B12 | *Erwinia billingiae* | G | L | R | OP102604 |
| B13 | *Erwinia* sp. | G | L | R | OP102605 |
| B14 | *Phyllobacterium myrsinacearum* | G | L | R | OP102606 |
| B15 | *Bacillus mycoides* | G | T | R | OP102607 |
| B16 | *Curtobacterium flaccumfaciens* | G | T | R | OP102608 |
| B17 | *Paraburkholderia hospita* | G | T | R | OP102609 |
| B18 | *Paraburkholderia hospita* | G | T | R | OP102610 |
| B19 | *Bacillus aryabhattai* | G | L | E | OP102611 |
| B20 | *Paenibacillus* sp. | G | T | E | OP102612 |
| B21 | *Pseudomonas* sp. | G | T | E | OP102613 |
| B22 | *Erwinia billingiae* | G | T | E | OP102614 |
| B23 | *Pantoea agglomerans* | G | T | E | OP102615 |
| B24 | *Erwinia* sp. | G | T | E | OP102616 |
| B25 | *Erwinia billingiae* | G | T | E | OP102617 |
| B26 | *Erwinia aphidicola* | G | LT | E | OP102618 |
| B27 | *Bacillus megaterium* | G | L | E | OP102619 |
| B28 | *Bacillus megaterium* | G | L | E | OP102620 |
| B29 | *Erwinia billingiae* | G | LT | R | OP102621 |
| B30 | *Acinetobacter* sp. | G | LT | R | OP102622 |
| B31 | *Rhizobium* sp. | G | LT | R | OP102623 |
| B32 | *Pseudomonas* sp. | G | T | E | OP102624 |
| B33 | *Caballeronia arationis* | G | T | R | OP102625 |
| B34 | *Bacillus megaterium* | G | L | E | OP102626 |
| B35 | *Erwinia billingiae* | G | T | E | OP102627 |
| B36 | *Pantoea* sp. | G | T | E | OP102628 |
| B37 | *Pseudomonas sp.* | S | LT | E | OP102629 |
| B38 | *Cedecea* sp. | G | LT | R | OP102630 |
| B39 | *Enterobacteriaceae bacterium* | G | LT | R | OP102631 |
| B40 | *Dyella japonica* | S | LT | R | OP102632 |
| B41 | *Erwinia* sp. | G | LT | R | OP102633 |
| B42 | *Paenarthrobacter* sp. | G | L | R | OP102634 |
| B43 | *Curtobacterium* sp. | G | LT | R | OP102635 |
| B44 | *Caballeronia glathei* | G | LT | R | OP102636 |
| B45 | *Rhizobium* sp. | G | L | R | OP102637 |
| B46 | *Paraburkholderia hospita* | G | LT | R | OP102638 |
| B47 | *Rahnella aquatilis* | S | LT | R | OP102639 |
| B48 | *Erwinia* sp. | G | T | E | OP102640 |
| B49 | *Pseudomonas* sp. | S | LT | E | OP102641 |
| B50 | *Pantoea agglomerans* | G | T | E | OP102642 |
| B51 | *Paraburkholderia caledonica* | G | LT | R | OP102643 |
| B52 | *Ralstonia* sp. | G | LT | R | OP102644 |
| B53 | *Erwinia* sp. | G | LT | R | OP102645 |
| B54 | *Ralstonia* sp. | G | LT | R | OP102646 |
| B55 | *Erwinia* sp. | G | LT | R | OP102647 |
| B56 | *Paraburkholderia* sp. | G | LT | R | OP102648 |
| B57 | *Rahnella aquatilis* | S | L | E | OP102649 |
| B58 | *Rhizobium* sp. | G | LT | E | OP102650 |
| B59 | *Bacteroidetes bacterium* | G | LT | R | OP102651 |
| B60 | *Rahnella* sp. | S | LT | E | OP102652 |
| B61 | *Lelliottia amnigena* | S | T | E | OP102653 |
| B62 | *Rahnella* sp. | S | L | E | OP102654 |
| B63 | *Pseudomonas graminis* | S | LT | E | OP102655 |
| B64 | *Rahnella aquatilis* | S | LT | E | OP102656 |
| B65 | *Serratia* sp. | S | LT | E | OP102657 |
| B66 | *Staphylococcus epidermidis* | S | LT | E | OP102658 |
| B67 | *Pseudomonas salomonii* | S | LT | E | OP102659 |
| B68 | *Bacillus* sp. | S | LT | R | OP102660 |
| B69 | *Paraburkholderia ginsengisoli* | S | LT | R | OP102661 |
| B70 | *Pseudomonas* sp. | S | LT | R | OP102662 |
| B71 | *Pseudomonas* sp. | S | LT | R | OP102663 |
| B72 | *Rahnella aquatilis* | S | LT | R | OP102664 |
| B73 | *Phyllobacterium ifriqiyense* | S | LT | R | OP102665 |
| B74 | *Phyllobacterium* sp. | S | LT | R | OP102666 |
| B75 | *Erwinia* sp. | G | LT | E | OP102667 |
| B76 | *Mitsuaria* sp. | G | LT | E | OP102668 |
| B77 | *Curtobacterium* sp. | G | T | R | OP102669 |
| B78 | *Pseudomonas* sp. | S | T | E | OP102670 |
| B79 | *Pseudomonas* sp. | S | T | E | OP102671 |
| B80 | *Lelliottia amnigena* | S | T | E | OP102672 |
| B81 | *Erwinia* sp. | S | T | R | OP102673 |
| B82 | *Erwinia* sp. | S | T | R | OP102674 |
| B83 | *Erwinia* sp. | S | T | R | OP102675 |
| B84 | *Enterobacteriaceae bacterium* | G | LT | R | OP102676 |
| B85 | *Rhizobium lusitanum* | G | LT | R | OP102677 |
| B86 | *Enterobacteriaceae bacterium* | S | L | E | OP102678 |
| B87 | *Lelliottia* sp. | S | T | E | OP102679 |
| B88 | *Serratia* sp. | S | LT | E | OP102680 |

**References**

Albuquerque, L., França, L., Rainey, F.A., Schumann, P., Nobre, M.F. and da Costa, M.S., 2011. Gaiella occulta gen. nov., sp. nov., a novel representative of a deep branching phylogenetic lineage within the class Actinobacteria and proposal of Gaiellaceae fam. nov. and Gaiellales ord. nov. *Systematic and applied microbiology*, *34*(8), pp.595-599.

Cao, L., Qiu, Z., You, J., Tan, H. and Zhou, S., 2004. Isolation and characterization of endophytic Streptomyces strains from surface‐sterilized tomato (Lycopersicon esculentum) roots. *Letters in applied microbiology*, *39*(5), pp.425-430.

Cătălina, S., Catalina, E.R. and Maria, R.C., 2019. Biodegradation of synthetic dyes by some bacterial strains isolated from soil. *Lucrări Ştiinţifice, Universitatea de Ştiinţe Agricole Şi Medicină Veterinară" Ion Ionescu de la Brad" Iaşi, Seria Horticultură*, *62*(1), pp.201-208.

Cherif-Silini, H., Silini, A., Yahiaoui, B., Ouzari, I. and Boudabous, A., 2016. Phylogenetic and plant-growth-promoting characteristics of Bacillus isolated from the wheat rhizosphere. *Annals of Microbiology*, *66*(3), pp.1087-1097.

Egamberdieva, D., 2012. Colonization of Mycobacterium phlei in the rhizosphere of wheat grown under saline conditions. *Turkish Journal of Biology*, *36*(5), pp.487-492.

Gao, J.L., Lv, F.Y., Wang, X.M., Yuan, M., Li, J.W., Wu, Q.Y. and Sun, J.G., 2015. Flavobacterium endophyticum sp. nov., a nifH gene-harbouring endophytic bacterium isolated from maize root. *International Journal of Systematic and Evolutionary Microbiology*, *65*(Pt_11), pp.3900-3904.

Hara, S., Morikawa, T., Wasai, S., Kasahara, Y., Koshiba, T., Yamazaki, K., Fujiwara, T., Tokunaga, T. and Minamisawa, K., 2019. Identification of nitrogen-fixing Bradyrhizobium associated with roots of field-grown sorghum by metagenome and proteome analyses. *Frontiers in microbiology*, *10*, p.407.

Jog, R., Pandya, M., Nareshkumar, G. and Rajkumar, S., 2014. Mechanism of phosphate solubilization and antifungal activity of Streptomyces spp. isolated from wheat roots and rhizosphere and their application in improving plant growth. *Microbiology*, *160*(4), pp.778-788.

Khamna, S., Yokota, A., Peberdy, J.F. and Lumyong, S., 2009. Antifungal activity of Streptomyces spp. isolated from rhizosphere of Thai medicinal plants. *International Journal of Integrative Biology*, *6*(3), pp.143-147.

Luo, X., Sun, X., Huang, Z., He, C., Zhao, J., Xiang, W., Song, J. and Wang, X., 2021. Actinoplanes flavus sp. nov., a novel cellulase-producing actinobacterium isolated from coconut palm rhizosphere soil. *International Journal of Systematic and Evolutionary Microbiology*, *71*(9), p.004990.

Madhaiyan, M., Poonguzhali, S., Lee, J.S., Lee, K.C. and Sundaram, S., 2010. Flavobacterium glycines sp. nov., a facultative methylotroph isolated from the rhizosphere of soybean. *International Journal of Systematic and Evolutionary Microbiology*, *60*(9), pp.2187-2192.

Mawdsley, J.L. and Burns, R.G., 1994. Inoculation of plants with a Flavobacterium species results in altered rhizosphere enzyme activities. *Soil Biology and Biochemistry*, *26*(7), pp.871-882.

Okoro, C.K., Bull, A.T., Mutreja, A., Rong, X., Huang, Y. and Goodfellow, M., 2010. Lechevalieria atacamensis sp. nov., Lechevalieria deserti sp. nov. and Lechevalieria roselyniae sp. nov., isolated from hyperarid soils. *International journal of systematic and evolutionary microbiology*, *60*(2), pp.296-300.

Roh, S.G., Lee, C., Kim, M.K., Kang, H.J., Kim, Y.S., Kim, M.J., Malik, A. and Kim, S.B., 2020. Nocardioides euryhalodurans sp. nov., Nocardioides seonyuensis sp. nov. and Nocardioides eburneiflavus sp. nov., isolated from soil. *International Journal of Systematic and Evolutionary Microbiology*, *70*(4), pp.2682-2689.
